# Characterization and phylogenetic analysis of the complete chloroplast genome of *Asterothamnus centraliasiaticus* Novopokr. (Asteraceae: *Asterothamnus*)

**DOI:** 10.1080/23802359.2024.2306207

**Published:** 2024-01-24

**Authors:** Zhengsheng Li, Ying Liu, Yushou Ma

**Affiliations:** Qinghai University, Qinghai Academy of Animal and Veterinary Sciences, Qinghai Provincial Key Laboratory of Adaptive Management on Alpine Grassland, Key Laboratory of Superior Forage Germplasm in the Qinghai-Tibetan Plateau, Xining, Qinghai, China

**Keywords:** *Asteraceae*, *Asterothamnus centraliasiaticus*, complete chloroplast genome, phylogenetic analysis

## Abstract

*Asterothamnus centraliasiaticus* Novopokr., a species of perennial deciduous semi-shrub within the family Asteraceae, has excellent medical, economic, ecological and genetic value. In this study, the chloroplast genome of *A. centraliasiaticus* was first assembled using Illumina HiSeq2500 sequences. The results indicate that the complete cp genome of *A. centraliasiaticus* is 152,205 bp in length, and comprises a pair of inverted repeat (IR) regions of 25,031 bp each, a large single-copy (LSC) region of 83,956 bp and a small single-copy (SSC) region of 18,187 bp. The GC content of *A. centraliasiaticus* is 37.3%. A total of 130 genes were successfully annotated containing 85 protein-coding genes, 37 transfer RNA genes, and 8 ribosomal RNA genes. The maximum likelihood (ML) phylogenetic analysis based on the complete chloroplast genome data highly supported that *A. centraliasiaticus* was close to *Aster lavandulifolius*. These results will provide significant genetic information for the germplasm protection and reasonable development.

## Introduction

**Figure 1.**
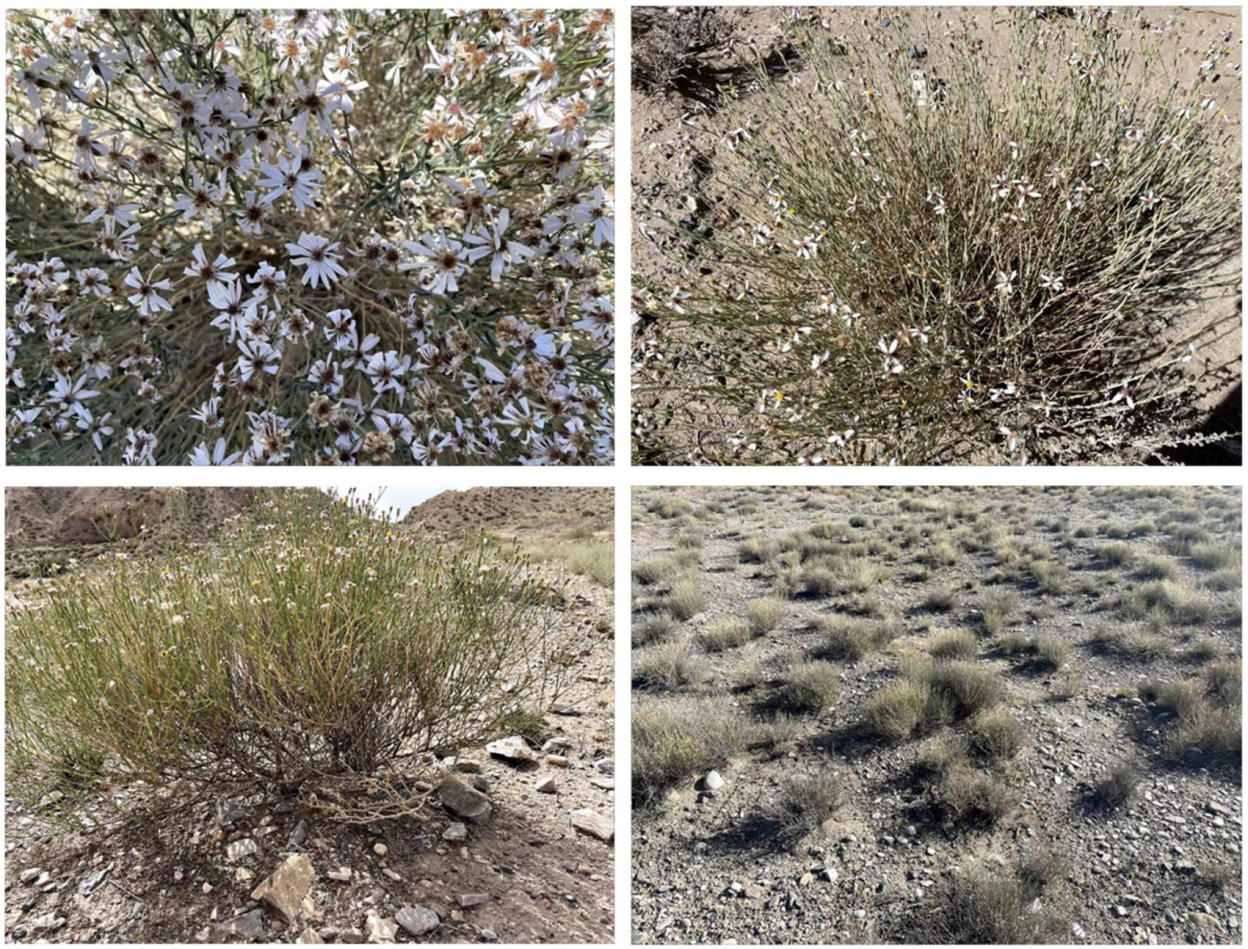
Morphology and habitat map of *Asterothamnus centraliasiaticus*. From yueliang Bay Park in Guide County, Hainan Tibetan Autonomous Prefecture, Qinghai Province, China (101°53’47" E, 36°09’23" N, 2050 m a.s.l.), photograph by Zheng-sheng Li. *A. centraliasiaticus* is a much-branched semi-shrub with plants up to 120 cm. Its stems are clustered, much branched below, with inflorescence branches above. Leaves crowded, ascending or erect, linear-oblong to linear, (8–)12–15 × 1.5–2 mm, abaxially densely gray-white villous, adaxially gray green, moderately to sparsely villous, base attenuate, margin revolute, apex acute. Capitula radiate 8–10 × ca. 10 mm, in loose corymbiform synflorescences or solitary at ends of branches; peduncles rather thick, straight or curved; bracts small, linear. Involucre broadly campanulate, 6–7 × ca. 9 mm; phyllaries 3- or 4-seriate, imbricate, white villous to arachnoid, glandular at least distally, margin broadly scarious, erose, apex acuminate to acute or ± obtuse, usually purplish red, outer short, ovate or lanceolate, inner oblong. Ray florets 7–13, tube ca. 2.9 mm, glabrous, lamina pale purple to pale lilac, 10–12 × 1.2–2.7 mm; disk florets 11–19(–26), yellow turning reddish, ca. 5 mm, tube 1.8–2.5 mm, tube and limb base sparsely hairy, limb narrowly campanulate to funnelform, ca. 2.7 mm. Achenes straw-colored, 3–3.5 mm, white strigose. Pappus white or pale cinnamon.

*Asterothamnus centraliasiaticu*s Novopokr 1950 (Novopokrovsky 1950), is a species of perennial deciduous semi-shrub within the family Asteraceae, and is natively distributed in arid and semi-arid areas of the Qinghai-Tibetan Plateau in China and Northwest Mongolia, naturally occurring at elevations of 1300–3900 m (Figure 1). *A. centraliasiaticus* is an ecologically important species within its range especially by functioning in soil stabilization and as a food resource for livestock and wildlife (Chai et al. 2010). Furthermore, *A. centraliasiaticus* possesses bright-colored flowers, and can also be exploited as ornamental plant. However, this valuable wild germplasm has been seriously threatened by human activities and climate changes during the past decades, and need urgent conservation (Chai et al. 2011). A good insight into its population genetics would be fundamental to formulating efficient strategies for its conservation, management and exploitation (Yang et al. 2018). Unfortunately, to date there are no reports of complete chloroplast (cp) genomes for this species or even for plants of the same genus. To contribute to such efforts, we reconstructed its complete chloroplast genome from high-throughput illumina sequencing data and analyzed its phylogenetic position within Asteraceae based on 20 cp genome sequences. This study will provide significant genetic information for the germplasm protection and reasonable development.

## Materials and methods

The fresh leaves of a single individual were collected from Guide County, Hainan Tibetan Autonomous Prefecture, Qinghai Province, China (101°53′47" E, 36°09′23" N, 2050 m a.s.l.), in August 2022. After on-site collection, the samples were immediately flash-frozen in −80 liquid nitrogen and then directly mailed to the sequencing company (Biotechnologies Inc.). The specimens (voucher no. QHGN-YLW24A5) have been deposited in the Key Laboratory of Superior Forage Germplasm in the Qinghai-Tibetan Plateau. College of Qinghai Academy of Animal and Veterinary Sciences, Qinghai University, Xining, China (URL: https://mky.qhu.edu.cn/index.htm, contact person: Ying Liu and email is liuying_yanhong@sina.com).

The total genomic DNA was extracted from leaf tissues using the TruSeq DNA sample Preparation kit (Vanzyme, China), and then sequenced using the Illumina Hiseq 2500 platform (Illumina, SanDiego, CA) with paired-end reads of 150 bp by Genesky Biotechnologies Inc, Shanghai, China. In total, 32,019,330 raw reads (2 × 150 bp) were obtained, which were trimmed using FastQC v 0.11.8. After trimming, the high-quality paired-end reads were assembled through metaSPAdes software (version 3.13.0). The sequence was matched to the genome, and all positions read mapping depth of assembled genome was statistically obtained, respectively. In the process, cp genome of *Aster indicus* (GenBank: NC040126) was used as reference genome, and the genome annotation was performed with the program by CPGAVAS2 software (version N/A) (Shi et al. 2019) comparing the sequences with the cp genome of *A. indicus*. The map of cis/trans-splicing gene and plastome using the online program CPGView (http://www.1kmpg.cn/cpgview) (Liu et al. 2018), and then the annotated cp genome sequence was submitted to the GenBank with the accession number OP909739.

The complete chloroplast sequences of *A. centraliasiaticus*, and other thirteen species in *Aster* were used in the phylogenetic analysis. Two *Medicago* species were used as outgroups. All of the 16 cp genome sequences were obtained from GenBank, and were aligned using MEGA7 with default parameter. The maximum likelihood (ML) tree was built using MEGA7 with bootstrap set to 1,000.

## Results

**Figure 2.**
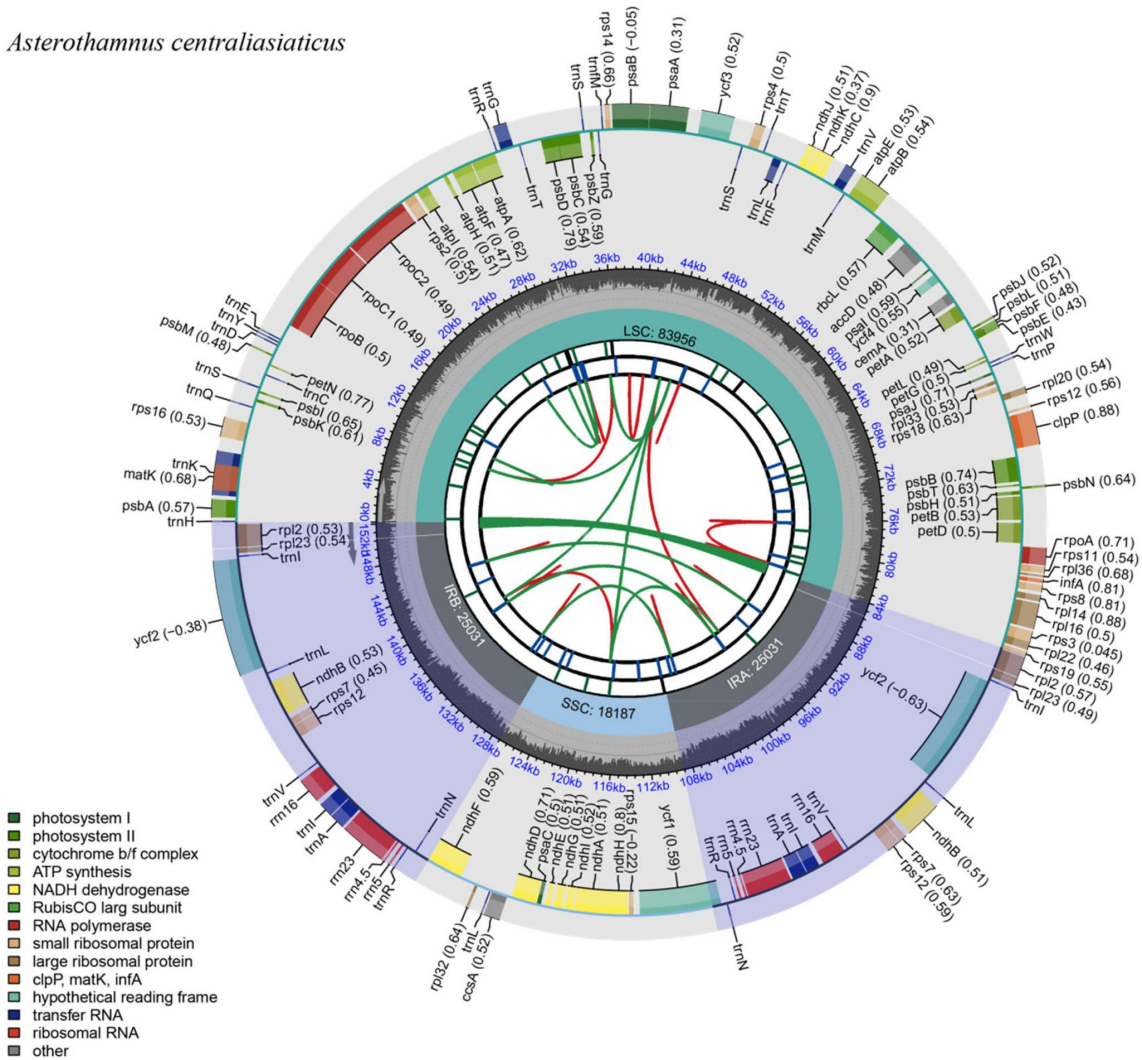
Chloroplast genome map of *Asterothamnus centraliasiaticus*. The species name is shown in the left top corner. From the center outward, the first track shows the dispersed repeats. The dispersed repeats consist of direct (D) and palindromic (P) repeats, connected with red and green arcs. The second track shows the long tandem repeats as short blue bars. The third track shows the short tandem repeats or microsatellite sequences as short bars with different colors. The colors, the type of repeat they represent, and the descriptions of the repeat types are as follows. Black: c (complex repeat); green: p1 (repeat unit size = 1); yellow: p2 (repeat unit size = 2); purple: p3 (repeat unit size = 3); blue: p4 (repeat unit size = 4); orange: p5 (repeat unit size = 5); red: p6 (repeat unit size = 6). The small single-copy (SSC), inverted repeat (IRa and IRb), and large single-copy (LSC) regions are shown on the fourth track. The GC content along the genome is plotted on the fifth track.

The results indicate that the maximum and average read mapping depths for assembled genomes were ×3320 and ×378 (Figure S1), respectively (Figure S1). The complete cp genome of *A. centraliasiaticus* is 152,205 bp in length (Figure 2), and comprises a pair of inverted repeat (IR) regions of 25,031 bp each, a large single-copy (LSC) region of 83,956 bp and a small single-copy (SSC) region of 18,187 bp. The GC content of *A. centraliasiaticus* is 37.32%. A total of 130 genes were successfully annotated containing 85 protein-coding genes, 37 transfer RNA genes, and 8 ribosomal RNA genes. Among these genes, 11 genes (rsp16, rpoC1, atpF, ycf3, clpP, petB, petD, rpl16, rp12, ndhB, and ndhA) are cis-spliced, and rps12 is also a trans-spliced gene (Figure S2). Additionally, 21 genes (*rps*12*, rps*16*, rpo*C1*, atp*F*, pet*B*, pet*D*, rpl*16*, rpl*2*, ndh*B*, ndh*A*, trn*K-UUU*, trn*G-UCC*, trn*L-UAA*, trn*V-UAC*, trn*A-UGC *and trn*I-GAU) have one intron each, and two genes (*ycf*3 *and clp*P) contain two introns.

**Figure 3.**
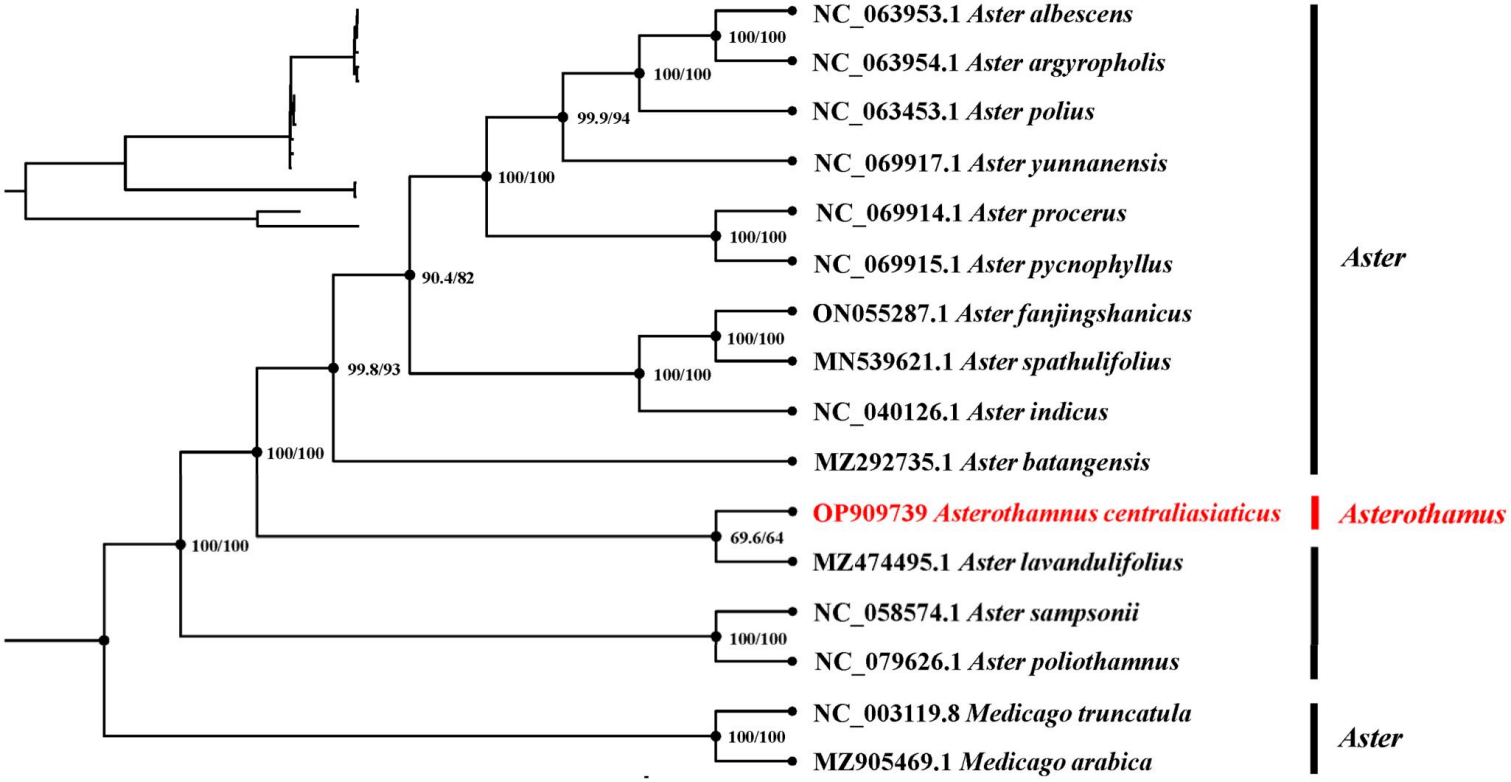
Chloroplast phylogeny of *Asterothamnus centraliasiaticus* based on the complete chloroplast genome sequences. The numbers on each branches indicate the boot support value of the ML analyses. The clades of species are represented with black lines. The following sequences of each species were used: *Aster albescens* NC063953.1, *Aster argyropholis* NC063954, *Aster polius* NC063453, *Aster yunnanensis* NC069917, *Aster procerus* NC069914.1, *Aster pycnophyllus* NC069915.1, *Aster fanjingshanicus* ON055287.1, *Aster spathulifolius* MN539621.1 (Choi and Park 2015), *Aster indicus* NC040126.1, *Aster batangensis* MZ292735 (Xie et al. 2021), *Aster lavandulifolius* MZ474495, *Aster sampsonii* NC058574.1 (Chang et al. 2021), *Aster poliothamnus* NC0796264.1, *Medicago truncatula* NC003119.8, *Medicago arabica* MZ905469.1 (Jiao et al. 2022). Undescribed citations in the legend indicate that the citations have not been published.

The complete chloroplast sequences of *A. centraliasiaticus*, and other thirteen species in *Aster* were used in the phylogenetic analysis. Two *Medicago* species were used as outgroups. Phylogenetic analysis indicated that *A. centraliasiaticus* was close to *Aster lavandulifolius* (Figure 3).

## Discussion and conclusion

As a result of sequencing chloroplast genomes from a variety of plants, we have gained a deeper understanding of chloroplast biology, biodiversity conservation (Teske et al. 2020), and genetic information that can be used to enhance agronomic traits in plants or to produce high value agricultural or biomedical products (Daniell et al. 2016). In this study, the chloroplast genome of *A. centraliasiaticus* was first assembled using Illumina HiSeq2500 sequences. The results indicate that the complete cp genome of *A. centraliasiaticus* is 152,205 bp in length. Similar to previous studies (Kwon et al. 2022; Liu et al. 2018), it is also a standard quadripartite structure, comprises a pair of inverted repeat (IR) regions of 25,031 bp each, a large single-copy (LSC) region of 83,956 bp and a small single-copy (SSC) region of 18,187 bp. A total of 130 genes were successfully annotated containing 85 protein-coding genes, 37 transfer RNA genes, and 8 ribosomal RNA genes. In addition, the sequence of the chloroplast genome has been widely used to determine evolutionary relationships in plants (Liu et al. 2022; Yang et al. 2018). In this study, the maximum likelihood (ML) phylogenetic analysis based on the complete chloroplast genome data highly supported that *A. centraliasiaticus* was close to *Aster lavandulifolius.* The obtained results will yield substantial genetic data pertaining to germplasm preservation and rational advancement. Furthermore, our findings indicate that certain species within the *Aster* genus exhibit a greater degree of dissimilarity at the chloroplast genome level compared to *A. centraliasiaticus*. This discovery necessitates a reassessment of the botanical species classification of *A. centraliasiaticus,* which in turn will furnish novel perspectives and theoretical substantiation for subsequent plant taxonomy.

## Supplementary Material

Supplemental Material

## Data Availability

The genome sequence data that support the findings of this study are openly available in GenBank of NCBI at https://www.ncbi.nlm.nih.gov under the accession no. OP909739. The associated BioProject, SRA, and Bio-Sample numbers are PRJNA906027, SRR22436051, and SAMN31890561, respectively.

## Plant material collection statement

The plant materials were not obtained from nature reserves, and the chloroplast genome sequencing research did not affect the population, so collection licenses were not needed.
