# Supplementary material for: Characterization and phylogenetic analysis of the complete chloroplast genome of Asterothamnus centraliasiaticus Novopokr. (Asteraceae: Asterothamnus)
Source: Mitochondrial DNA B Resour. 2024 Jan 24;9(1):168–72. doi: 10.1080/23802359.2024.2306207 (PMC10810623; doi:10.1080/23802359.2024.2306207)
Supplement: Supplemental Material [file TMDN_A_2306207_SM4631.docx]

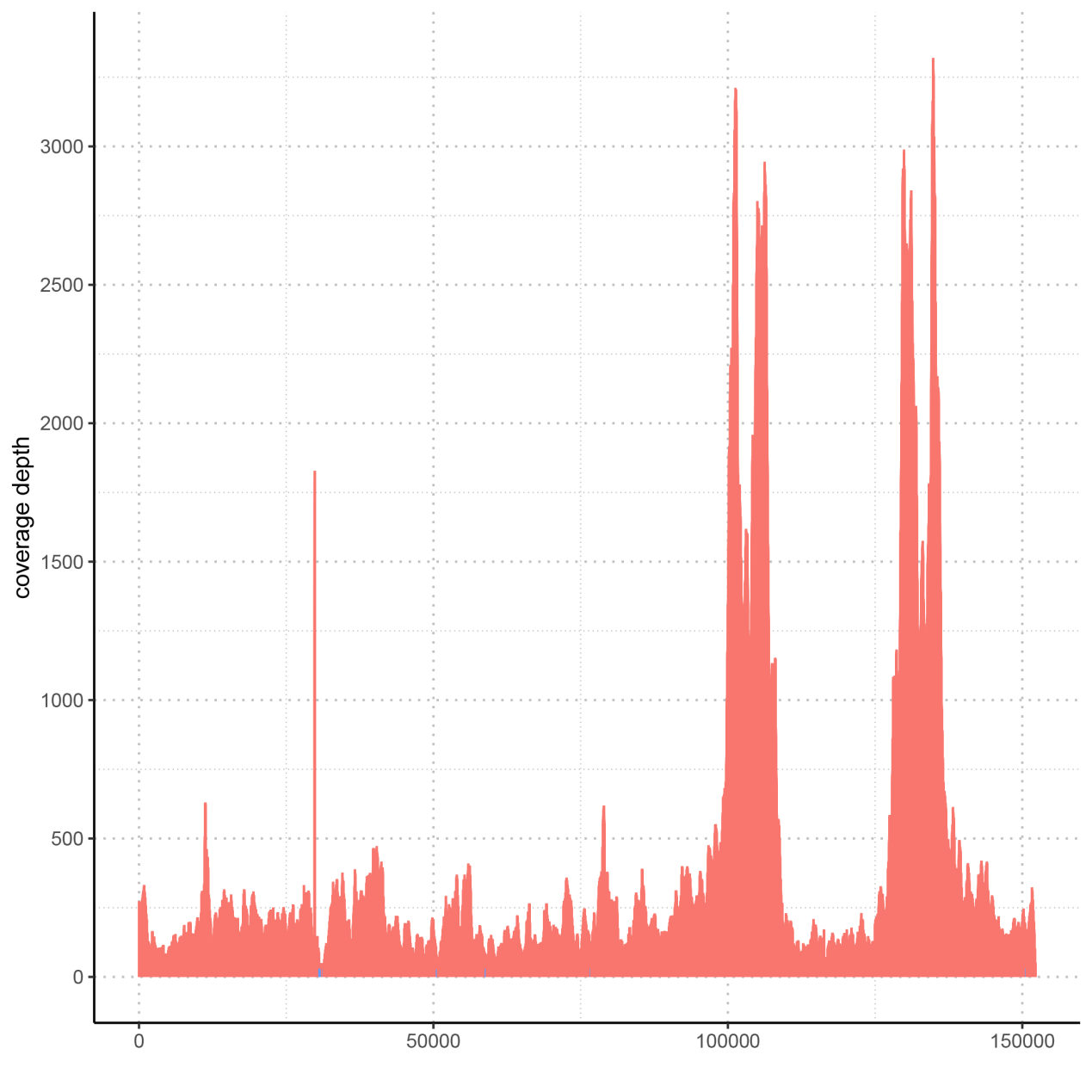


**Figure S1 Read mapping depth of the plastome sequence.** Illumina read mapping depth is presented with red bars. X and Y axis present nucleotide position of plastome and read mapping depth, respectively.


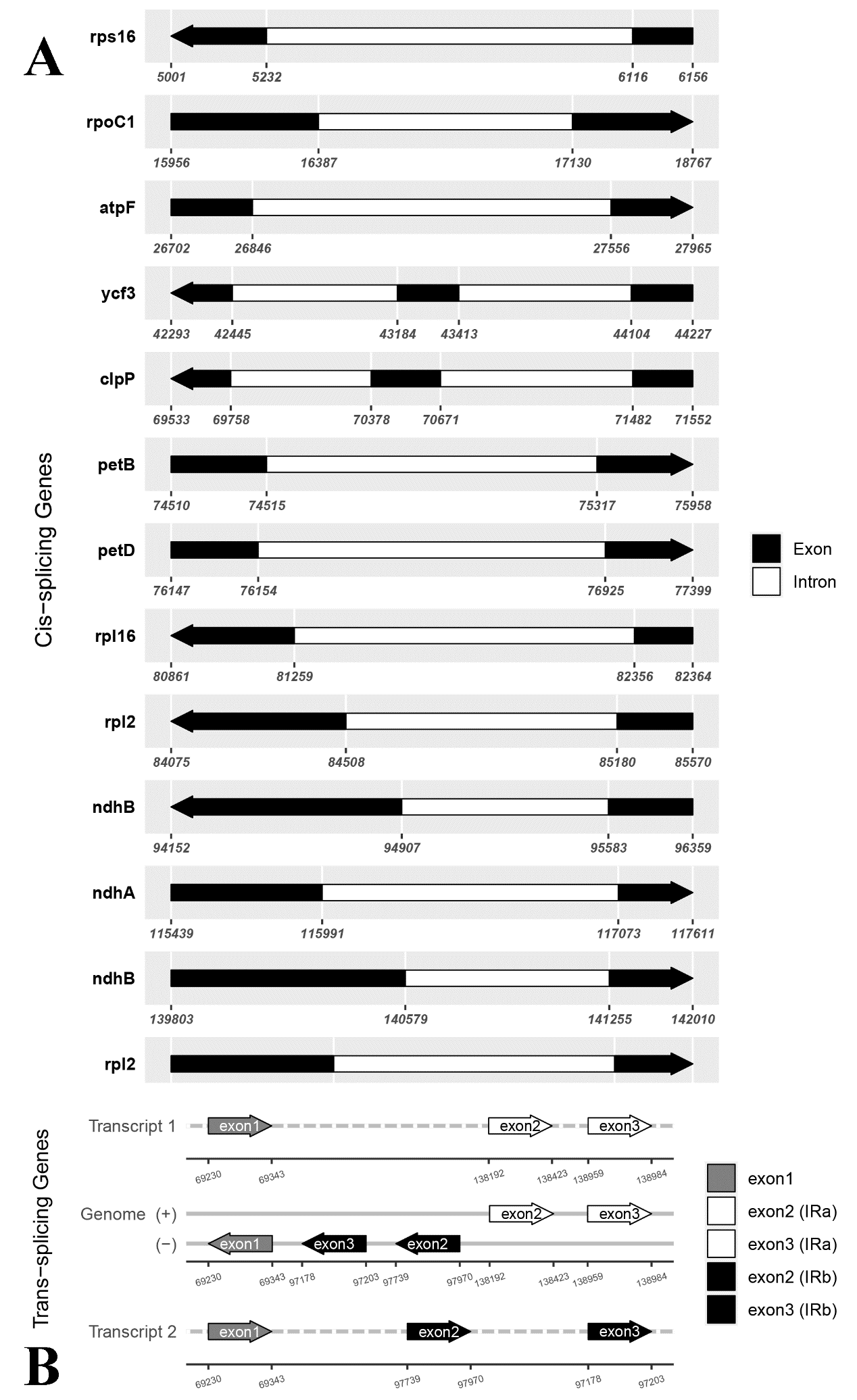
**Figure S2 The map of cis-splicing and trans-splicing gene.** A. Schematic map of the cis-splicing genes in the *A. centraliasiaticus* chloroplast genome. B. Schematic map of the trans-splicing gene rps12 in the chloroplast genome.
